# Prevalence of Typhoid and Paratyphoid Fever in the Hohoe Municipality of the Volta Region, Ghana: A Five-Year Retrospective Trend Analysis

**DOI:** 10.5334/aogh.2833

**Published:** 2020-09-03

**Authors:** Adam Fusheini, Sarfo Kofi Gyawu

**Affiliations:** 1 Department of Preventive and Social Medicine, Dunedin School of Medicine, University of Otago, NZ; 2 Center for Health Literacy and Rural Health Promotion, GH; 3 Department of Epidemiology and Biostatistics, School of Public Health, University of Health and Allied Sciences, GH

## Abstract

**Background:**

Typhoid and paratyphoid fever remain a global public health burden, yet annual estimates of prevalence vary. Estimates have ranged between 9.9 and 24.2 million cases annually. Similar differences in estimates are seen within countries but point to a serious health challenge. In Ghana, for instance, typhoid fever has been ranked among the top twenty causes of outpatient morbidity and constituted 1.2%, 1.7% and 1.3% of hospital admissions in 2017, 2016 and 2015 respectively.

**Objective:**

The objective of the study was to determine the prevalence of *Salmonella Typhi* and *Salmonella Paratyphi* in the Hohoe Municipality.

**Methods:**

Data on all reported cases of typhoid fever in the Hohoe municipality as entered into the District Health Information Management System 2 (DHIMS 2) database between January 2012 and December 2016 were extracted. A time-trend analysis was conducted to establish the relationship between typhoid fever prevalence and factors such as age, gender, and season. Stata was used to analyse data and to measure rates, associations, and their significance.

**Findings:**

The results showed that a total of 6282 individuals suffered from typhoid fever during the five-year period. Of these numbers, 2080 (33.1%) were males, and 4202 (66.9%) were females, representing a P-value 0.0222, and 95% CI. The 25–29 age group were the most affected. High prevalence was observed during the wet months, although cases occurred throughout the year. Trend analysis showed growing cases of typhoid over the period. Prevalence for the various years were found as follows: 2012 – 148 per 100,000, 2013 – 135 per 100,000, 2014 – 396 per 100,000 and in 2015 – 943 per 100,000.

**Conclusions:**

Typhoid fever remains and continues to be a major public health challenge in the municipality. This calls for health authorities and service providers to educate the public about the disease if the challenge is to be addressed.

## Introduction

Typhoid and paratyphoid fevers are infections caused by bacteria, which are transmitted from feces to ingestion. *Salmonella enterica* serovars Paratyphi A, B, and C, cause the clinically similar condition, paratyphoid fever [1]. *Salmonella* is classified into two major groups: the invasive and the non-invasive. The invasive *Salmonella*, otherwise called typhoidal *Salmonellae* causes enteric fevers [2]. Typhoidal *Salmonellae* include *Salmonella* Typhi and Paratyphi. The non-invasive *Salmonella* are termed non-typhoidal *Salmonellae* (NTS) and are made up of *Salmonella* species, which usually cause gastroenteritis [2]. Typhoid and paratyphoid fevers are collectively referred to as enteric fevers, which pose a considerable burden to about 5.5 billion people living in low- and middle-income countries (LMICs) [3]. Typhoid and paratyphoid fevers are caused by related but different strains of *Salmonella spp, and there* is considerable overlap in symptoms. Typhoid has the likelihood of resulting in death without prompt treatment. Untreated enteric fever has a case fatality rate of 10% or higher [4], while typhoid fever has a case fatality rate of 10%–30% [5] without effective treatment. Again, it is important to mention that some *Salmonella* serovars are host-specific. *Salmonella* Typhi and *Salmonella* Paratyphi *A, B*, and *C* are associated with humans [6,7].

Meanwhile, there are differences in global data estimates of typhoid fever, but all point to a worrying situation of dramatic increases in Sub-Saharan Africa [8,9]. For instance, typhoid fever has been estimated by some studies to cause between 9.9 and 24.2 million cases and 75,000–208,000 deaths per year [3,10,11,12]. Another study put the global burden of typhoid fever to between 21.7 million illnesses and 217000 fatalities annually [1]. Thus, salmonella infection accounts substantially for the global burden of morbidity and mortality [1,13,14], especially in LMICs. In most endemic areas, approximately 90% of enteric fever is typhoid. An important risk factor for typhoid fever is lack of or no access to clean and safe water [11], and is commonly found among crowded and impoverished populations with poor and inadequate sanitation [7]. Typhoid fever is, therefore, associated with poor, unhygienic and inadequate sanitary conditions and poor access to clean portable water [15].

While efforts to quantify the global burden of enteric fever are valuable for understanding the health lost and the large-scale spatial distribution of the disease [16], typhoid fever prevalence data are scarce, particularly in LMICs [3]. This often results in varying estimates of annual prevalence. For instance, the WHO has estimated the incidence of typhoid to be about 17 million cases worldwide (WHO 2016). Meanwhile, in a systematic review published in 2019, researchers estimated that 14.3 million cases of typhoid and paratyphoid fevers occurred in 2017 of which Salmonella *enterica* serotype Typhi caused 76.3% of cases of enteric fever with an estimated 135.9 thousand deaths from typhoid and paratyphoid fever globally in 2017 [16]. Estimating prevalence is, therefore, a major problem, which has implications in the attempt to reduce the incidence. This, notwithstanding, the Typhoid Fever Surveillance in Africa Program (TFSAP) has helped in providing new incidence figures for typhoid fever in sub-Saharan Africa [1,17]. The difficulty in estimating the prevalence is partly due to the considerable overlap in symptoms of typhoid and paratyphoid, although typhoid is more severe and lasts longer.

Approximately, 2%–5% of the population infected with *S. Typhi* become chronic carriers, which is usually higher among women and among persons with biliary abnormalities such as gallstones [18]. The spread of drug-resistant and increase in the incidence of non-Typhi salmonella, particularly in sub-Saharan Africa and in patients co-infected with HIV, implies that there is now more than ever a need for patient-oriented clinical research in enteric fever [19]. An earlier study shows the highest incidence of *Salmonella* cases worldwide occurs in South East Asian and Latin American countries of about 274 cases per 100,000 persons and 50 cases per 100,000 persons [20], respectively. The population at high risk of typhoid infection was about 1.6 billion (29%), whereas the remainder 4·0 billion were at risk [11].

In Sub Saharan Africa, including Ghana, there is little data to describe typhoid or paratyphoid fever case-fatality rates. In a study on the global typhoid fever burden, Crump et al. assumed a case-fatality rate of 1% for typhoid fever based on hospital-level data, expert opinion, and mortality rates documented by advanced surveillance systems [21]. The wrong diagnosis of typhoid cases as malaria adds to the complexity of fatality figures. The true magnitude is difficult to quantify because the clinical picture is confused with many other febrile illnesses, and most typhoid endemic areas lack facilities to confirm the diagnosis. The prevalence of Salmonella infection in many parts of sub-Saharan Africa is largely unknown. This is attributed to the lack of diagnostic laboratories with fatal Salmonella oftentimes attributed to malaria [22]. Indeed, in Ghana, the burden of typhoid fever and its associated complications have been reported in various prior studies. This, notwithstanding, the latest reported cases for typhoid fever in Ghana still showed a worrying trend and a major public health concern. In 2017, 2016 and 2015, 365,148, 384,704, and 337,120 cases of typhoid fever were recorded and ranked among the top twenty causes of outpatient morbidity and 1.2%; 1.7% and 1.3% of hospital admissions respectively [23]. This buttressed and corroborated a prior study where clinical and laboratory data coalesced for 15 years (1975–1990) from four African countries (Ghana, Zambia, Tanzania, and Kenya) showed that Ghana had a national incidence of one in a thousand, the highest among the countries surveyed [24,25]. *Salmonella* infections in Ghana is ranked among the leading 20 causes of outpatient illness, accounting for 0.92% of hospital admissions in 2008 [26]. Although, the etiologies of typhoid fever and malaria are different, with the former caused by a bacterium, and the latter by a protozoan and transmitted via different mechanisms, both diseases share rather similar symptomatology [27]. Individuals in endemic areas for both diseases are at substantial risk of contracting both, either concurrently or an acute infection superimposed on a chronic one because both diseases share social circumstances, which are important to their transmission [5].

In another study conducted between September 2007 and November 2008 at the Agogo Presbyterian Hospital in Ghana, it was revealed that of 1,456 children < 15 years of age who were admitted to the pediatric ward of the hospital over the 23-month study period, the rate of typhoid fever was 0.7% among children two years and above. This increased to 7% among children aged 2 to 11 and plummeted to 4.6% among children older than 11 years [28]. Also, the incidence of typhoid fever was highest in children 2–5 years of age, which was 290 per 100,000. The researchers noted that despite the high incidence that was recorded, rates might be higher due to the low sensitivity of microbiology methods to *Salmonella* infections. The conclusion was that more work was needed in the surveillance for typhoid fever, improvement of diagnostic methods to obtain more and reliable data in Ghana and West Africa concerning typhoid fever, and introduction of a vaccination program for the disease.

Similarly, in a study of the prevalence of Salmonella infections among patients admitted at the St Dominic Hospital in Akwatia, Eastern Region, the researcher looked at cases, in general, using microbiology diagnostic methods to classify the isolated serovars. In 464 patients’ samples that were collected consisting of 194 blood samples and 270 stool samples for bacteriological culture, typhoidal salmonellae were isolated from 29.6% of these samples of which 20.4% was due toSalmonella Typhi and 9.3% Salmonella Paratyphi B [25]. Non-typhoidal *Salmonellae* was 70.4%, which represented the total of the other isolates from all the other samples.

A related study conducted at the Paediatric Unit of the Komfo Anokye Teaching Hospital in Kumasi noted that out of the 296 children that had Salmonella bacteremia, 87.2% was due to *Salmonella spp* and 12.8% was due to *Salmonella typhi* [29]. This highlights a high prevalence in the country, and underscores the significance of this study as to the best of our knowledge; there is currently no work on typhoid fever prevalence in the Hohoe Municipality. Yet, information from the District Health Information Management System (DHIMS 2) shows that in 2015, 1575 cases of typhoid fever were recorded in the Hohoe Municipality, indicating a high prevalence in the area. It is against this background that this study was conducted with the overall aim of determining the prevalence of *Salmonella typhi* and *Salmonella Paratyphi* in the Hohoe Municipality from the health records of persons reporting at the Municipal health facilities between 2012 and 2016. In other words, we sought to determine a five-year trend of typhoid fever prevalence in the area. Specifically, however, the study addressed the following four research objectives. Firstly, to determine the yearly incidence of *Salmonella Typhi a*nd *Salmonella Paratyphi* species among people in the Hohoe Municipality. Secondly, to determine the prevalence of *Salmonella Typhi* and *Salmonella Paratyphi* in all patients who were treated or admitted at the Municipal Hospital. The third objective was to determine any associations and relationships between age, gender, and time of year with Typhoid fever prevalence; and, finally, to determine the period of the prevalence of typhoid fever from January 2012–December 2016.

The anticipation is that this study would provide empirical baseline information to the Hohoe Municipal Health Directorate regarding the prevalence rate of typhoid fever in the district based on hospital or facility reported and or confirmed cases. This could then lead to the implementation of efficient public health policies and programs aimed at combating the problem. Knowledge of the prevalence can also be used by other areas in Ghana and other sub-Saharan African countries in designing policies and programs aimed at improving and promoting population health and wellbeing.

## Materials and Methods

### Study Design

Cross-sectional study design was adopted with data collection covering patients’ records from the health facilities in the municipality between January 2012 and December 2016. This design ensured we collected the data at one point in time to enable our description of the pattern of prevalence of typhoid fever. Our objective was to obtain a structured set of data that enabled systematic comparisons between the incidence over the five year period [30]. We needed data on typhoid fever cases from the various years to be able to describe the situation using the same variables. As our interest was to compare the cases/incidence of typhoid and to account for variations between cases in terms of pattern and prevalence, a cross-sectional design was considered the most appropriate [30].

### Study Setting

The study was conducted in the Hohoe municipality, which is one of the then twenty-five administrative districts in the Volta Region. The 2010 population and housing census put the population at 167,016, with 79,967 males and 87,049 females. The total land area is 1,172km sq, which represents 5.6% of the region’s landmass. The municipality lies at the heart of the region bordering about five other areas. These include the Republic of Togo on the East, Afadzato South district on the South East, the Kpando Municipality to the South West, Jasikan district on the north, and the Biakoye District on the northwest. The rains start in late April and end by October. Temperatures are high during the year and range from 26 degrees Celsius in the coolest months to 32 degrees Celsius in the hottest months. Factors such as the sanitation, which is not so good in the area, use of stored water, and washing with contaminated water make people more susceptible to food and water-borne diseases.

### Study Population

The study population included all persons who reported to any of the health facilities in the municipality to be treated for Salmonella between January 2012 and December 2016. The sampling frame for the study was, therefore, the number of cases of typhoid that had been recorded during the period.

### Exclusion and Inclusion Criteria

The study included anyone who all the various health facilities in the municipality were able to isolate either Salmonella Typhi or Salmonella Paratyphi from either his or her blood or stool sample from January 2012 to December 2016. People who never visited the health facilities during the period or visited but were not treated for typhoid or paratyphoid fever were excluded. People suspected of typhoid but with no lab confirmation were also be excluded.

### Sampling Method

Purposive sampling was used to target and focus on only confirmed typhoid fever cases and for the period of the study. The sample size depended on the number of cases recorded in the DHIMS 2 database for all the sub-districts under the municipality. This was to ensure the representativeness of the study.

### Data Collection Procedure

Data used for the study were based on the records of the health directorate of the Hohoe Municipality. Access to the data was facilitated by the University, providing an introductory letter to the Hohoe Municipal Health Directorate requesting the data for the period January 2012 to December 2016. All health facilities in the municipality were included in the study. The Municipal Health Information Officer extracted data on all typhoid fever cases during the designated period from the DHIMS 2 database. The extracted data were made available in an electronic format to the second author. All typhoid fever cases from January 2012 to December 2016 were extracted and used for the study.

### Data Analysis

The extracted data were entered into Microsoft Excel 2016. The data were then exported to STATA version 12.0 for analysis, including measuring rates and associations. Two sample test of proportions was used to determine factors associated with typhoid fever prevalence. 95% confidence interval was estimated, and a p-value of < 0.05 was considered statistically significant.

### Ethical Issues

Data collected did not include personal or identifying information. The study received approval from the Ethical Review Committee (ERC) of the Ghana Health Service, Ministry of Health (GHS/MOH), with approval number GHS-ERC 53/10/16C.

## Results

The results of the data extracted from patient records in DHIMS 2 in line with the objectives of the study are presented below.

### Characteristics of study participants

A total of 6282 people were found to have suffered from typhoid fever during the study period. Out of this number, 2080 (33.1%) were males, and 4202 (66.9%) of the study participants were females. Typhoid fever cases were high in females compared to males in all but one age group. Among all patients’ data taken, the 25–29 age group recorded the highest number of cases, with 9.4% of all cases, followed by 20–24. The age group with the least number among the participants was 0–11 months (1.6%). The second-lowest was amongst 30–34 (4.8%). In males, the highest rates were recorded in the 1–4 age group, which produced 50.3% of all cases in that age group. The lowest for males was the 65–69 age group, which recorded 21.8 for that age range. Among females, the 50–59 age group was the highest, at 79.7%. The 1–4 age group (49.7%) recorded the lowest number of cases for females (Table 1).

**Table 1:** Demographic characteristics of participants.

Year	Male	Proportion	Female	Proportion	Total	Zscore	P-value

0–11 months	43	2.07	55	1.31	98	2.286	0.022
1–4	223	10.72	220	5.24	443	7.983	<0.001
5–9	183	8.80	247	5.88	430	4.313	<0.001
10–14	190	9.13	257	6.12	447	4.367	<0.001
15–19	177	8.51	337	8.02	514	0.667	0.505
20–24	206	9.90	341	8.12	547	2.355	0.019
25–29	171	8.22	419	9.97	590	–2.238	0.025
30–34	116	5.58	186	4.43	302	2.005	0.045
35–39	137	6.59	382	9.09	519	–3.387	0.001
40–44	88	4.23	135	3.21	223	2.057	0.040
45–49	111	5.34	351	8.35	462	–4.302	<0.001
50–54	79	3.80	311	7.40	390	–5.565	<0.001
55–59	114	5.48	316	7.52	430	–3.014	0.003
60–64	64	3.08	148	3.52	212	0.560	0.576
65–69	53	2.55	190	4.52	243	–3.811	0.000
70+	125	6.01	307	7.31	432	–1.916	0.055

Using the two-sample test of proportions, the analysis was done to check the significance of differences between the various age groups and gender with typhoid fever. This was done by comparing the number of cases in males and females. All but three of the age groups showed that differences were statistically significant. These were the 15–19, 60–64, and 70+, age groups.

### The yearly incidence of Salmonella Typhi and Salmonella Paratyphi species among people in the Hohoe Municipality

**Figure 1:**
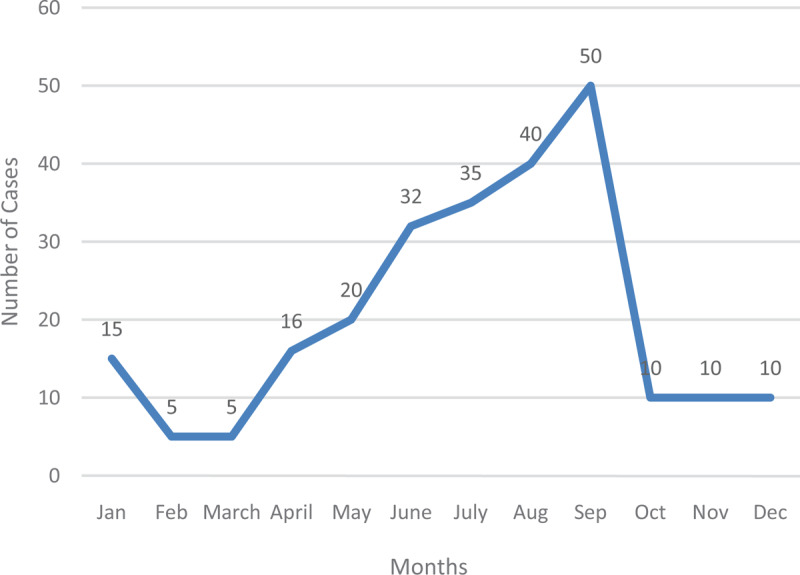
Typhoid Cases in 2012.

Analysis of diagnosis of typhoid fever did not specify if Salmonella Typhi or Paratyphi caused them. Due to similarities in signs, only laboratory confirmation can distinguish between the two. Unfortunately, the secondary data collected from DHIMS 2 were not aggregated to differentiate between the two. The yearly incidence of the disease, as extracted from DHIMS 2 are presented below (Figure 1).

**Figure 2:**
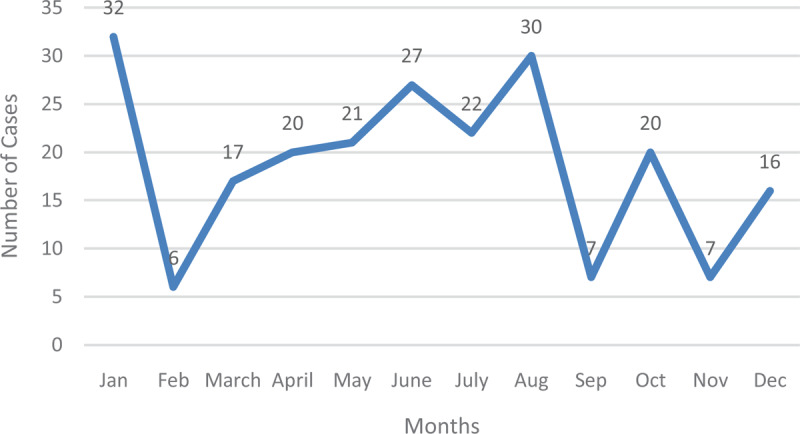
Typhoid Fever in 2013.

In 2012, the lowest number of cases recorded in the Hohoe Municipality was in February and March (5). September in that year recorded 50 cases. Generally, cases recorded during this year were low. January recorded 15 cases, which reduced in February by 10. The same number was recorded in March. From there, cases gradually increased month by month, until it reached a peak in September. A sharp decrease occurred from September, and cases remained at ten new cases per month until the year ended (Figure 2).

**Figure 3:**
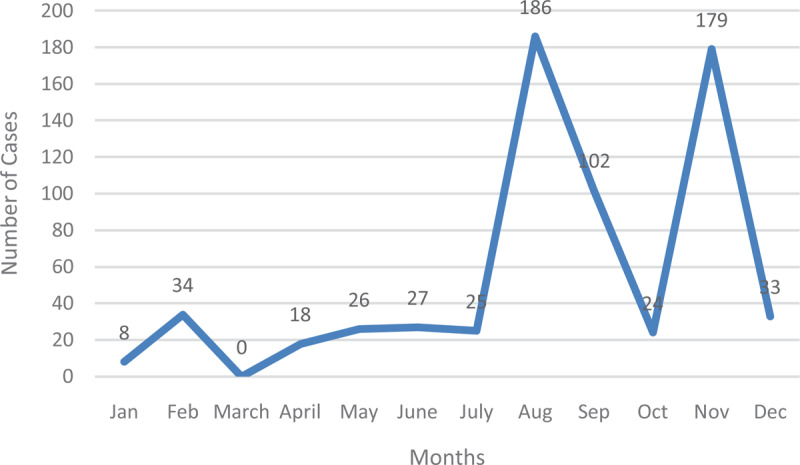
Typhoid Fever Cases in 2014.

The figure below represents the number of typhoid cases in the year 2013 with January recording the highest, followed by August with September and November producing the lowest number of cases. The year 2013 was in sharp contrast to the previous year since the disease peaked in January, and recorded the lowest in the following month. Subsequent months recorded fluctuations in the number of typhoid fever cases until the turn of the year. August produced the second-highest number of typhoid fever cases (Figure 3).

**Figure 4:**
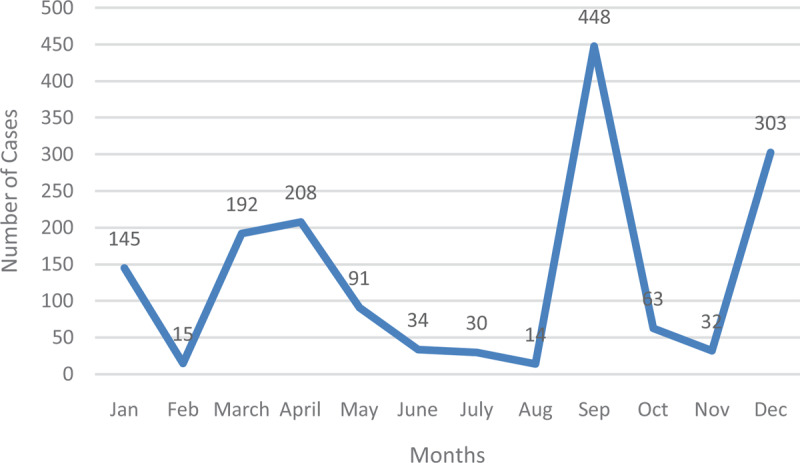
Typhoid Fever Cases in 2015.

In 2014, cases gradually increased before peaking in August, reducing slightly in September and recording the second-highest in November (Figure 4).

**Figure 5:**
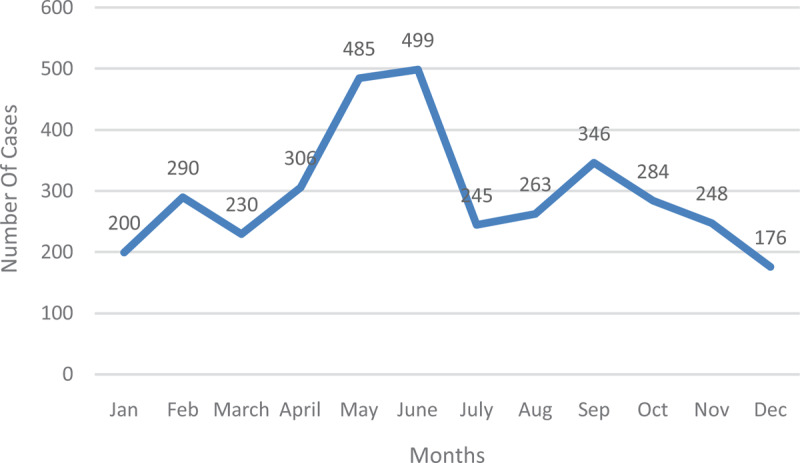
Typhoid Fever Cases in 2016.

In 2015, cases began at 145 in January before witnessing a sharp drop in February. After March and April, numbers gradually decreased. A peak was recorded in September, which reduced in the ensuing months before the year ended with 303 (Figure 5).

**Figure 6:**
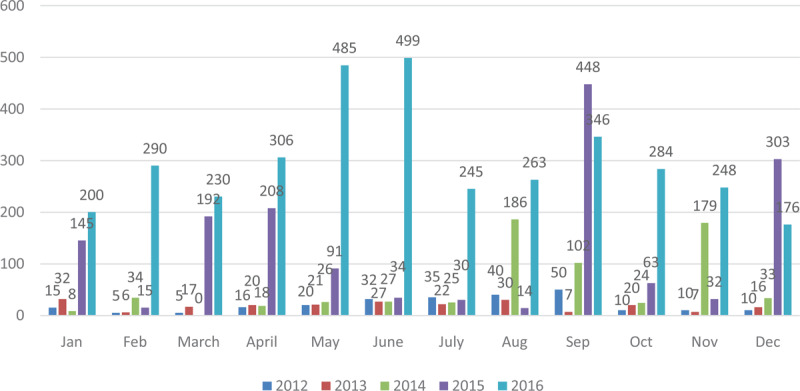
Typhoid Fever Cases from January 2012–December 2016.

**Figure 7:**
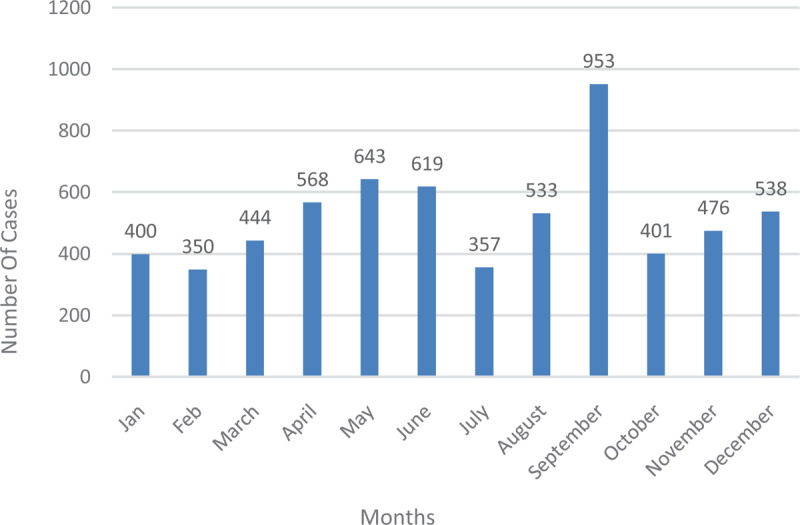
Typhoid fever cases by months during the study period.

The year 2016 saw a fluctuation in the incidence of typhoid fever over the twelve-month period. The lowest incident of typhoid fever was recorded at the end of the year in December (176) and the highest in the middle of the year in June (499). June is often the peak of the rainy season in the municipality (Figures 6 and 7).

The figure below shows the cumulative sum of typhoid fever cases in the various months from January to December 2012–2016. Cases recorded were highest in September and lowest in February.

### Associations between age, gender, season with typhoid fever prevalence and rates during the study period

**Figure 8:**
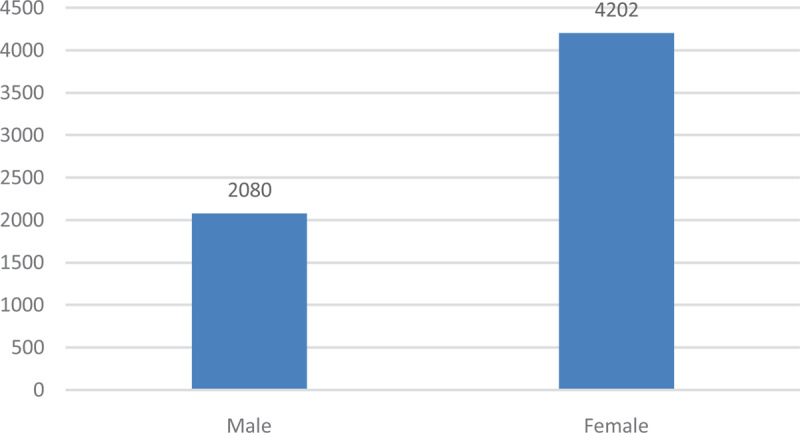
Typhoid fever cases by Gender.

The Figure 8 shows typhoid cases in males and females during the study period.

There were high rates in females compared with males. This was investigated using a 2-sample proportion test. Using a 95% confidence interval, the P-value was found to be 0.0222 and the z-score 2.2864. This meant that there was a difference between typhoid in males and females, which was statistically significant (Table 2).

**Table 2:** Typhoid Fever Cases among Various Age Groups (Male).

Age	Male					

	2012	2013	2014	2015	2016	Total

0–11 months	1	4	1	26	11	43
1–4	8	5	10	96	104	223
5–9	8	3	23	37	112	183
10–14	13	10	10	45	122	190
15–19	12	7	27	32	106	177
20–24	6	12	32	25	143	206
25–29	9	12	15	52	95	171
30–34	11	6	19	50	36	116
35–39	5	2	15	27	90	137
40–44	8	2	10	20	50	88
45–49	13	3	16	28	54	111
50–54	6	3	4	28	41	79
55–59	3	4	6	20	85	114
60–64	6	7	2	16	40	64
65–69	2	1	5	15	31	53
70+	6	6	13	31	75	125
Total	**117**	**87**	**208**	**548**	**1195**	**2080**

Among males, typhoid fever cases were highest amongst the 20–24 year group, followed by the 25–29 age group. The lowest numbers were recorded in the 0–11 month category. The cases increased year after year with the exception of 2013, which recorded a drop from its preceding year (Table 3).

**Table 3:** Typhoid Fever among Various Age Groups (Females).

Age	Female					

	2012	2013	2014	2015	2016	Total

0–11 months	3	3	0	37	12	55
1–4	5	3	14	100	98	220
5–9	9	10	37	67	124	247
10–14	6	7	31	50	163	257
15–19	24	14	21	79	199	337
20–24	8	20	50	123	140	341
25–29	12	16	64	120	207	419
30–34	6	4	34	40	102	186
35–39	7	6	34	84	251	382
40–44	6	12	12	50	55	135
45–49	8	10	30	53	250	351
50–54	9	7	69	65	161	311
55–59	10	9	40	55	202	316
60–64	5	5	19	20	99	148
65–69	6	7	30	37	110	190
70+	7	12	18	74	196	307
Total	**131**	**145**	**503**	**1054**	**2369**	**4202**

The major difference between cases in males and females was in the 20–24 and 25–29 year groups. Cases were rather highest in the 25–29 year groups in females, followed by the 20–24 age group instead of vice versa in the cases of males. Generally, the trend was similar; 0–11 months recorded the lowest cases as in that of males.

### Period prevalence of typhoid fever from January 2012 to December 2016

**Figure 9:**
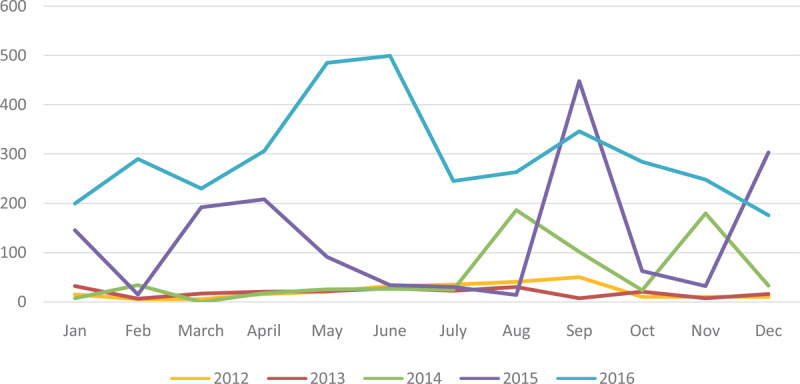
A trend analysis showing the prevalence of typhoid fever from January 2012 to December 2016.

Comparing the various years, 2013 recorded the lowest figures (Figure 9). It began to increase from 2014 through 2015 and 2016. Across all the years, the period from May–July and August–September produced the highest number of cases. Low numbers were recorded from November to March.

## Discussion

### The yearly incidence of **Salmonella typhi** and **Salmonella Paratyphi** species among people in the Hohoe Municipality

Since both species were not distinguished during the entry of data into DHIMS, typhoid fever cases were analysed as being caused by either of them. Out of the total of five years of the study, typhoid fever figures soared in 2016, followed by 2015, 2014, and then 2013. This shows an increasing rate of typhoid fever in Hohoe as the years go by, and from this, it could be predicted that that of 2017 and subsequent years could be higher if measures were not put in place to deal with it. A similar increase in Salmonella cases year by year was observed by another study [25]. Data from other typhoid fever studies show a fluctuating trend during study periods but usually point towards an increasing trend when significance analysis is done. From the average number of cases recorded on a yearly basis during the study period, 2012 recorded 20 cases per year, 2013 recorded 18.8. This sharply began to rise with 55.2 in 2014, 131.3 in 2015, and finally the highest in 2016, which was 297.5.

Malisa & Nyaki found these same fluctuations and increasing incidence in their work in Tanzania [5]. Mweu & English estimate of the global burden of enteric fever suggested a moderate incidence of typhoid of 10–100 cases/100,000 persons a year in most African countries [31]. Neil et al. give the annual typhoid fever incidence in Africa as 13 to 845 cases per 100 000 population [32]. Data from this study also show an increasing trend in cases of typhoid year after year. Without risk factor correction, Mogasale et al. revealed that West Africa had a typhoid fever incidence of 213 per 100,000 [11]. In that study, Ghana was identified as a high-risk region because she recorded more than 100 cases per 100,000 yearly, thereby confirming the results from the study.

### Period prevalence of typhoid fever from January 2012 – December 2016

In calculating the prevalence, the cue has been taken from the work of Mogasale et al., which used a population per 100,000 to calculate prevalence [11]. This is also done in studies by Buckle et al. and Crump [10,21] and many others. Data from the 2010 population and housing census estimates the total population of the Hohoe Municipality as 167,016, of which 79,967 representing 47.9% are males, and 87,049 representing 52.1% are females [33]. Using this as the base population during the study period and calculating the prevalence by cases recorded in each of the study years gives the following.

2012 – 148 per 100,000
2013 – 135 per 100,000
2014 – 396 per 100,000
2015 – 943 per 100,000
2016 – 2,139 per 100,000

It should, however, be noted that these calculations were done assuming that the population size remains constant or reaches equilibrium when all other factors are considered, with no adjustments made to cater for growth rate and fatality rate in the municipality.

### Associations between age, gender, and time of year with typhoid fever prevalence

Most of the typhoid fever cases were recorded in the 25–29 age range. The most susceptible group is often thought of as children under five, but this is inconclusive. Most works on typhoid fever give different age ranges and class intervals, which makes the confirmation of very susceptible age groups nearly impossible. Neil et al. reported the age group highest hit as the 5–19-year age group [32]. Crump et al. had earlier indicated the age group of 5–9 as being those who suffer most while those within the age bracket of 20–24 as those who suffer the least from it [13]. These differences in age range are observed in other studies of typhoid fever. While one study asserts that the 26–35 year age group displayed preponderance over the others [34], another study in two different locations in Kenya-Lwak and Kiberia points to the 18–34 and the 5–9 age groups respectively as being affected most [35].

It is also established in this study that females were the most diagnosed population group with typhoid fever. Similar findings have been noted in common Salmonella infections in a hospital in Akwatia in the Eastern region of Ghana, where females represented 55.6% [25]. Outside Ghana, studies of typhoid fever have revealed high rates of Salmonella Typhi prevalence in females. For instance, in Benue state central in Nigeria, Salmonella Typhi prevalence was found to be 58.0% [36]. This does not, however, suggest that other researchers have not found contrary trends in relation to gender and prevalence. In a research published in the Clinical Infectious Diseases Journal in 2012, typhoid fever prevalence was found to be highest among males (59.0%) in a study conducted in the Kasese District of Uganda from 2008–2009 [32]. The high prevalence among females maybe because they engage in household chores like fetching of water, washing, and cooking. We assume that the water used might be infested with the Salmonella bacterium. Additionally, the prevalence could be attributed to the irregular flow of tap water in Hohoe, resulting in people resorting to well water and the purchase of stored water from vendors. Added to the above is the absence of toilet facilities in most homes due to the association and occurrence of the disease in places with low hygiene and poor sanitary conditions.

The findings regarding the peak months in this study are consistent with prior studies. From the data presented earlier, typhoid fever cases were generally high during the third quarter of the year, followed by the number of cases recorded in the second quarter of the year and lowest during the first quarter. High rates coincided with the rainy season with low ones during the dry season. The peaks of March, April, November, July, and September for different years were also observed in a study conducted at the Korle-Bu Teaching hospital in Accra [37]. The highest values during the third quarter of the year were also found in Tanzania [5]. Outside sub-Saharan Africa, studies on paratyphoid fever in India demonstrate evidence of recorded high cases during the monsoon season [38]. Notwithstanding, typhoid fever cases are recorded all year round, and changes in the weather have little or no impact on its rate as each month recorded cases.

## Limitations

There were a number of limitations encountered during the course of the study, including the following. One major limitation was the difficulty in specifying from the extracted data of health records which cases were caused by paratyphoid serovars as all recorded cases were captured under typhoid fever. This is because data in DHIMS do not often specify if Salmonella Typhi or Paratyphi caused typhoid cases. However, since both species and their subtypes and strains can be classified as suffering from typhoid fever, this does not affect the quality of the study. Another limitation is that not all people infected with typhoid went to the health facilities for treatment, which might affect the true values of the disease prevalence. Other limitations were the possibility of misdiagnosis of some typhoid fever cases as other diseases and vice versa; wrong entry of typhoid fever data from the health facilities, and lastly, data were aggregated; therefore, regression tests and other tests could not be run to check for other associations. The results of the study should, therefore, be interpreted with this in mind.

## Conclusion

Typhoid fever remains very high and continues to be a worrying public health challenge in the Hohoe Municipality. Data from all the sub-districts in the municipality as extracted from DHIMS2 point still to an increasing trend, with the 25–29 age group being the most at risk. Health authorities and service providers have the responsibility to educate the public about the symptoms and consequences of typhoid in the municipality, given the high prevalence. This is crucial since initial symptoms of the disease are similar to malaria and could be mistaken for malaria by the public. Consequently, failing to report early enough to health facilities could lead to health-threatening conditions like perforated intestines, anemia, and hepatomegaly, among others, which are fatal.
